# Evaluation of the impact of reducing fasting hours on laboratory results in a mediterranean population

**DOI:** 10.11613/BM.2025.020703

**Published:** 2025-04-15

**Authors:** Gemma Solé-Enrech, Ruth Cano-Corres, Raquel Escribano-Tembleque, Eva Martínez-Sevilla, Núria Busquets-Carmona, Carlos García-Miralles

**Affiliations:** Clinical laboratory, Biochemistry Department, Parc Taulí Research and Innovation Institute Foundation (I3PT), Sabadell, Spain

**Keywords:** fasting hours, preanalytical phase, venous blood sampling

## Abstract

**Introduction:**

One of the critical points of the preanalytical phase is the patient’s adherence to the required fasting duration before undergoing medical analysis. Although many laboratories have already protocols for blood-sample collection that require only a 6-hour fast, clinical guidelines remain unclear on this aspect, and fasting periods of 12 hours are sometimes still recommended. The aim of this study was to evaluate whether there are significant differences between the results obtained in patients’ serum samples obtained post-fasting, 4 hours post-meal, and 6 hours post-meal for different predetermined parameters.

**Materials and methods:**

30 volunteers (16 females and 14 males) aged between 23 and 62 years were selected for this study. Each participant underwent an initial analysis after a 10-hour fast (baseline), a second analysis 4 hours after a controlled meal, and a third analysis 6 hours after the meal. The parameters studied correspond to previously selected biochemical, hematological, and coagulation tests. To assess if there are significant differences in the results obtained for each analyte, criteria based on the total allowable error (TEa) and the reference change value (RCV) were used.

**Results:**

All parameters evaluated in this study met the criteria based on the RCV at both 4 and 6 hours, although some parameters did not meet TEa criteria.

**Conclusions:**

The results obtained in this study demonstrated that a fasting period of 4 or 6 hours is sufficient to obtain reliable results. This could significantly improve the quality of life for patients undergoing analysis without compromising the quality of their results.

## Graphical abstract

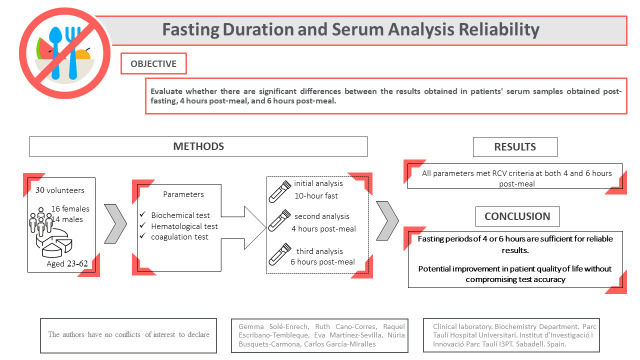

## Highlights

• Effect of fasting duration on the measurement of 27 biochemical markers, complete blood count, and two coagulation parameters was investigated

• Metrological and biological variability were used in statistical analysis

• The results obtained suggest the proposal of a reduced fasting time to 4 or 6 hours

## Introduction

The preanalytical phase is the stage preceding laboratory analysis and includes the period from when the doctor requests the analysis until the sample reaches the laboratory area and enters the analyzer where it will be processed. It involves patient preparation, sample collection, preservation, and administrative procedures. Processes within this phase require proper organization or management as they are a potential source of errors in the reported results. However, there is still a lack of standardization regarding patient preparation before conducting an analysis (*1*). This preparation includes items such as the fasting hours required, previous physical activity, tobacco and alcohol consumption, *etc.*

One of the critical points of this preanalytical phase is patient compliance with the fasting hours required to obtain reliable and quality results with no interference by food intake. Although many laboratories already have blood-sample collection protocols that require only a 6-hour fast, clinical guidelines still lack clarity on this aspect or recommend fasting periods of 12 hours (*1*-*3*).

The problem of waiting lists in healthcare not only affects outpatient consultations and surgery scheduling, but also laboratories, particularly for blood phlebotomies.

Due to increases in healthcare activity, modern laboratories now perform venous phlebotomies over a wide time frame (for example, our laboratory has extended blood collection hours until 2 p.m.). However, this strategy has a drawback of making it difficult for patients to comply with the necessary fasting hours, causing significant discomfort, and patients with delicate health conditions may even experience dizziness, nausea, and loss of consciousness.

Some countries have completely eliminated the fasting requirement to expand blood collection hours without negatively impacting patient’s quality of life (*4*). This practice has been criticized, but the reality is that there is a need to reduce fasting hours before conducting an analysis (*4*). Only then can laboratories respond to the increased demand experienced in recent years. The authors of this study have the hypothesis that 4 or 6 hours should be enough to guarantee the quality of results. For this reason, the aim of this study was to evaluate the effect of food intake on blood concentrations of biochemical parameters, coagulation factors and complete blood count as well as the possibility of reducing fasting hours to 4 or 6 hours post-meal.

## Materials and methods

The study involved 30 volunteers (16 women and 14 men) between 23 and 62 years of age who were employees of the Parc Taulí Healthcare Corporation in Sabadell. The health conditions of this volunteers were not assessed in order to reproduce the daily activity of the laboratory, which deals with patients with all types of pathology. Each participant was provided with an information sheet and signed an informed consent form. The study has been approved by the hospital’s clinical research ethics committee (Approval number: 2023/3021). Each participant underwent an initial venous venipuncture at 8 a.m. following a 10-hour fasting period (baseline). Immediately after this baseline venipuncture, a controlled meal was served to each participant. The meal provided consisted of a sandwich (with lettuce, tomato, tuna, and mayonnaise, 274 kcal/100g), a slice of yogurt cake (with chocolate 456 kcal/100g or without chocolate 394 kcal/100g), and juice (peach 26 kcal/100mL or pineapple 27 kcal/100mL). Each volunteer ingested approximately 350-550 kcal. A subsequent venous venipuncture was done 4 hours after the controlled meal and a final venous venipuncture 6 hours after the meal. The volunteers did not rest at any point.

From each volunteer and during every venipuncture, the following samples were collected: a 3.5 mL gel separator tube without anticoagulant (Vacuette CAT Serum Sep Clot Activator, Greiner Bio-One, Kremsmünster, Austria), a 3 mL EDTA plasma tube (Vacuette K3E K3EDTA, Greiner Bio-One, Kremsmünster, Austria), and a 3 mL citrate plasma tube (Vacuette 9NC Coagulation, 3.2% Sodium Citrate, Greiner Bio-One, Kremsmünster, Austria). All venipunctures were performed using tube holders (Greiner Bio-One, Kremsmünster, Austria). The EDTA and citrate plasma tubes were homogenized immediately after blood collection, while the serum samples were allowed to undergo clot retraction for 30 minutes. All venipunctures were carried out by the same experienced nurse.

Serum and citrate samples were centrifuged for 10 minutes at 1500xg using a CR3i multifunction centrifuge (Thermo Scientific, Waltham, USA) and processed immediately thereafter. Biochemical tests (serum) were performed using an automated Cobas 8000 system (c701, c502, and e801; Roche Diagnostics, Mannheim, Germany), while coagulation tests (citrate) were conducted using an ACL TOP 550 CTS system (Instrumentation Laboratory Co., Bedford, USA). Plasma samples (EDTA) were processed immediately after venipuncture using a Sysmex XN-9000 system (Sysmex, Kobe, Japan) for complete blood count analysis.

The analytes assessed were: albumin, alanine aminotransferase (ALT), alkaline phosphatase (ALP), aspartate aminotransferase (AST), total bilirubin (TBIL), calcium (Ca), cholesterol (CHOL), complete blood count, high-density lipoprotein cholesterol (HDL), low-density lipoprotein cholesterol (LDL), creatinine (CREA), ferritin, folate, glucose (Glc), gamma-glutamyltransferase (GGT), sodium (Na), potassium (K), iron (Fe), lactate dehydrogenase (LD), inorganic phosphate (Phos), c-reactive protein (CRP), total protein (TP), prothrombin time (PT), activated partial thromboplastin time (APTT), triglycerides (TG), thyroid-stimulating hormone (TSH), uric acid (UA), urea, vitamin B12 (vitB12) and vitamin D (vitD).

Complete blood count included: white blood cells, red blood cells, hemoglobin, hematocrit, mean corpuscular volume, mean corpuscular hemoglobin, mean corpuscular hemoglobin concentration, red cell distribution width, platelets, mean platelet volume, platelet distribution width, plateletcrit and neutrophils, lymphocytes, eosinophils, basophiles and monocytes count.

For each parameter of each sample, the hemolysis index, lipemia index, and icterus index were taken into account, and parameters affected by any of these indices were rejected.

All the magnitudes of the study met the specifications of imprecision and bias during the time it was conducted.

For each individual and each parameter, the differences between the results obtained at baseline and at 4 and 6 hours post-meal were calculated:

4h difference = (concentration (4h) - concentration (0h)) / concentration (4h) x 100
6h difference = (concentration (6h) - concentration (0h)) / concentration (6h) x 100.

Then the average of those individual differences was estimated. To check for any outlier result, the Dixon test was employed.

Two criteria were used to assess whether the differences obtained for each parameter are significant or not. The first criterion was based on the total allowable error (TEa), which takes into account the metrological characteristics of each technique. The TEa is defined on three ways:

1. In the quality-specification document issued by the Spanish Society of Laboratory Medicine (SEQC-ML) in 2023.
2. For parameters included in the complete blood count, TEa is obtained from the minimum consensus specifications (MCS) document of the scientific societies AEMB (Spanish Association of Medical Biopathology), AEFA (Spanish Association of Clinical Laboratory), SEHH (Spanish Society of Hematology and Hemotherapy), and SEQC published in 2017.
3. TEa is also defined in the databases of the European Federation of Clinical Chemistry and Laboratory Medicine (EFLM), which consider three categories: minimum, desirable, and optimal (*5*).

The second criterion considers the reference change value (RCV), which was calculated as follows: RCV = √2 x Z x √(CV_A_^2^ + CV_I_^2^), where CV_A_ is the analytical coefficient of variation obtained from internal quality control data, CV_I_ is the intra-individual coefficient of variation obtained from published biological variability tables (EFLM), and Z is a statistic that is equal to 1.96 in order to consider a significant change with 95% confidence (P < 0.05).

With each criterion, we obtained a value that cannot be exceeded by the average of the percentage difference as an absolute value obtained at 4 and 6 hours post-ingestion compared to the baseline value.

The data processing was done using an Excel spreadsheet (Microsoft office standard 2016).

## Results

Tables 1, 2, and 3 show the percentage average differences for each parameter and the different TEas. Table 1 includes the results of biochemical analytes, Table 2 includes the results of the complete blood count, and Table 3 includes the results of coagulation tests.

**Table 1:** Calculated RCV, different TEa and obtained percentage differences for biochemical analytes included in the study

**Analyte** **(unit)**	**Baseline (median and IQR)**	**RCV**	**TEa (SEQC) (%)**	**TEa (EFLM) minimum (%)**	**TEa (EFLM) desirable (%)**	**TEa (EFLM) optimal (%)**	**N1**	**4 h DIF (%)**	**N2**	**6 h DIF (%)**
Alb (g/L)	45.9 (44.34-47.80)	9.77	5.2	4.9	**3.3**	**1.6**	29	2.44	30	3.48
ALT (U/L)	14 (12-20)	29.2	8	28	18.7	9.3	30	4.66	30	6.09
ALP (U/L)	65 (55-89)	17.71	10.5	15.6	10.4	5.2	30	5.19	30	5.13
AST (U/L)	20 (17-21)	27.88	6.8	18.6	12.4	6.2	29	5.94	29	5.18
TBIL (µmol/L)	9.41 (6.84-10.43)	55.64	**12.4**	**36.9**	**24.6**	**12.3**	30	37.17	29	29.84
Ca (mmol/L)	2.42 (2.35-2.5)	6.84	3.4	3.4	2.3	**1.1**	30	2.18	30	1.78
CHOL (mmol/L)	5 (4.71-5.45)	15.66	4.3	12.5	8.3	4.2	30	2.83	30	3.29
HDL (mmol/L)	1.4 (1.18-1.85)	17.43	11.1	14.9	9.9	5	30	4	29	3.48
LDL (mmol/L)	2.96 (2.68-3.42)	23.8	-	-	-	-	28	6.1	28	3.86
CREA (mmol/L)	0.07 (0.06-0.08)	14.65	**7.4**	11.7	**7.8**	**3.9**	30	9.21	30	5.76
Ferritin (µg/L)	94.55 (38.33-186.53)	37.24	16.9	20.8	13.9	6.9	29	5.85	30	6.63
Folate (mmol/L)	10.4 (8.8-16.7)	33.45	19.7	23.3	15.5	**7.8**	29	11.22	27	9.11
Glc (mmol/L)	5 (4.77-5.39)	14.69	**6.5**	9.2	**6.1**	**3.1**	30	7.07	30	6.75
GGT (U/L)	14 (10-17)	25.81	9.4	27.5	18.3	9.2	29	7.28	28	6.53
Na (mmol/L)	139 (138-140)	3.09	1	0.9	**0.6**	**0.3**	29	0.84	30	0.77
K (mmol/L)	4.30 (4.19-4.40)	11.81	**2.4**	7.3	4.9	**2.4**	30	3.46	30	4.9
Fe (µmol/L)	15.84 (11.8-18.81)	57.45	**13.3**	48.6	32.4	**16.2**	30	14.55	30	27.1
LD (U/L)	155 (147-176)	15.95	7.7	10.2	6.8	**3.4**	29	5.91	29	4.55
Phos (mmol/L)	1.23 (1.09-1.33)	22.4	**4.9**	14.5	**9.6**	**4.8**	30	10.06	30	9.45
CRP (mg/L)	1.4 (0.88-1.83)	95.13	25.4	76.5	51	25.5	29	2.64	30	4.87
TP (g/L)	71 (69-74.1)	8.9	3.5	4.9	3.2	**1.6**	30	2.61	30	3.05
TG (mmol/L)	0.94 (0.79-1.34)	55.3	**13.5**	38.8	**25.9**	**12.9**	30	28.83	30	20.02
TSH (mIU/L)	1.885 (1.503-2.681)	50.09	**12.3**	37.3	**24.8**	**12.4**	30	34.09	30	36.73
UA (µmol/L)	306.32 (260.52-358.66)	23.66	**6.4**	19	12.6	**6.3**	30	5.20	30	6.9
Urea (mmol/L)	10.36 (9.18-13.68)	39.3	**8.9**	25.7	17.1	8.6	29	8.14	29	10.66
VitB12 (pmol/L)	282.65 (196.23-358.15)	22.9	12.7	23	15.4	7.7	30	5.17	30	6.47
VitD (nmol/L)	52.92 (42.88-67.59)	30.43	18.7	20	13.3	**6.7**	30	8.09	30	9.75
Alb - albumin. ALT - alanine aminotransferase. ALP - alkaline phosphatase. AST - aspartate aminotransferase. TBIL - bilirubin, total. Ca - calcium. CHOL - cholesterol. HDL - high density lipoprotein. LDL - low density lipoprotein. CREA - creatinine. Glc - glucose. GGT - gamma-glutamyltransferase. Na - sodium. K - potassium. Fe - iron. LD - lactate dehydrogenase. Phos - inorganic phosphate. CRP - C-reactive protein. TP - total protein. TG - triglycerides. TSH - thyroid stimulating hormone. UA - uric acid. VitB12 - vitamin B12. VitD - vitamin D. IQR - interquartile range. RCV - reference change value. TEa - total allowable error. SEQC - Spanish Society of Laboratory Medicine. EFLM - European Federation of Clinical Chemistry and Laboratory Medicine. N - number of data points used for the study after outliers were removed using the Dixon test (N1 at 4 hours and N2 at 6 hours). DIF (%) - percentage difference obtained at 4 and 6 hours post-ingestion compared to the baseline (fasting) result. The results exceeding TEa are presented in bold.										

**Table 2:** Calculated RCV, different TEa and obtained percentage differences for complete blood count

**Analyte (unit)**	**Baseline (median and IQR)**	**RCV**	**TEa (SEQC)**	**TEa (EFLM) minimum**	**TEa (EFLM) desirable**	**TEa (EFLM) optimal**	**N1**	**4 h DIF (%)**	**N2**	**6 h DIF (%)**
WBC (x 10^9^/L)	6.68 (5.52-7.52)	30.03	**10**	21.3	**14.2**	**7.1**	30	11.71	30	14.78
RBC (x 10^12^/L)	4.61 (4.47-4.89)	7.44	4.1	6.3	4.2	**2.1**	30	2.81	30	3.09
Hb (g/L)	137 (134.2-147.8)	7.71	4.6	5.8	3.9	**1.9**	29	2.54	30	2.41
Hct (L/L)	0.4 (0.4-0.41)	8.13	8.5	5.8	3.9	1.9	30	0.4	29	0.25
MCV (fL)	88.1 (86.12-90.75)	3.13	7.3	2.4	**1.6**	**0.8**	30	1.57	29	1.23
MCH (pg)	30 (29.1-30.85)	2.61	4.9	2.6	**1.7**	**0.9**	30	2.2	30	1.89
MCHC (g/L)	339 (333-344)	3.54	-	1.9	**1.3**	**0.6**	30	1.52	29	1.6
RDW (%)	12.6 (12.2-13.08)	4.98	-	-	-	-	29	1.09	30	1.33
Plt (x 10^9^/L)	250 (220-267)	21.46	16	15.3	10.2	5.1	29	4.03	30	4.41
MPV (fL)	10.6 (10.03-11.48)	6.93	-	5.6	3.8	**1.9**	30	2.71	30	3.67
PDW (fL)	12.4 (11.35-14.10)	11.50	-	-	-	-	30	4.68	30	7.47
Pct (L/L)	0.026 (0.023-0.028)	18.53	-	13.5	9	4.5	28	5.24	28	5.53
Neutrophils (x 10^9^/L)	3.24 (3.08-4.17)	39.30	**8.4**	27.6	18.4	**9.2**	30	15.95	30	19.98
Lymphocytes (x 10^9^/L)	2.16 (1.95-2.89)	30.35	19	22.8	15.2	**7.6**	30	13.19	30	14.53
Eosinophils (x 10^9^/L)	0.18 (0.11-0.265)	45.07	29	43.7	29.1	**14.6**	30	25.07	30	26.19
Basophils (x 10^9^/L)	0.04 (0.04-0.06)	35.19	100	26.2	**17.5**	**8.7**	30	18.42	30	21.12
Monocytes (x 10^9^/L)	0.52 (0.4-0.61)	38.30	73	26.2	17.4	**8.7**	30	12.32	30	10.84
WBC - white blood cells. RBC - red blood cells. Hb - hemoglobin. Hct - hematocrit. MCV - mean corpuscular volume. MCH - mean corpuscular hemoglobin. MCHC - mean corpuscular hemoglobin concentration. RDW - red cell distribution width. Plt - platelets. MPV - mean platelet volume. PDW - platelet distribution width. Pct - plateletcrit. IQR - interquartile range. RCV - reference change value. TEa - total allowable error. SEQC - Spanish Society of Laboratory Medicine. EFLM - European Federation of Clinical Chemistry and Laboratory Medicine. N - number of data points used for the study after outliers were removed using the Dixon test (N1 at 4 hours and N2 at 6 hours).). DIF (%) - percentage difference obtained at 4 and 6 hours post-ingestion compared to the baseline (fasting) result. The results exceeding TEa are presented in bold.										

**Table 3:** Calculated RCV, different TEa and obtained percentage differences coagulation tests included in the study

**Analyte (unit)**	**Baseline (median and IQR)**	**RCV**	**TEa (SEQC)**	**TEa (EFLM) minimum**	**TEa (EFLM) desirable**	**TEa (EFLM) optimal**	**N1**	**4 h DIF (%)**	**N2**	**6 h DIF (%)**
PT (s)	11.07 (10.73-11.52)	8.36	13	5.4	3.6	**1.8**	30	2.19	30	2.34
APTT (s)	33.5 (30.81-33.17)	9.67	12	6.4	**4.2**	**2.1**	29	3.33	28	4.35
PT - prothrombin time. APTT - activated partial thromboplastin time. IQR - interquartile range. RCV - reference change value. TEa - total allowable error. SEQC - Spanish Society of Laboratory Medicine. EFLM - European Federation of Clinical Chemistry and Laboratory Medicine. N - number of data points used for the study after outliers were removed using the Dixon test (N1 at 4 hours and N2 at 6 hours). DIF (%) - percentage difference obtained at 4 and 6 hours post-ingestion compared to the baseline (fasting) result. The results exceeding TEa are presented in bold.										

All three tables show cases where the percentage difference obtained compared to the fasting value exceeds the TEa in bold. For certain analytes TEa is not defined, such as LDL, RDW, and PDW. Therefore, this first criterion cannot be used for these analytes.

Tables 1, 2 and 3 also display the average percentage differences and the RCV calculated for each analyte. The tables show no case where the RCV was exceeded.

Taking into account the first criterion based on the TEa, the data obtained from this study shows that for the following magnitudes all TEa were met: ALT, ALP, AST, Ca, CHOL, HDL, ferritin, folate, GGT, CRP, B12, haematocrit, platelets, and eosinophils. But the following ones only met the minimum TEa: CREA, Glc, P, TG and TSH.

The rest of the evaluated magnitudes, except TBIL, met different TEa criterions in each case. At last, TBIL represents a special case because none of the TEa criterion was met.

For the second criterion, based on RCV, all magnitudes met the RCV proposed.

## Discussion

The results obtained in this study demonstrated that a fasting period of 4 or 6 hours is sufficient to obtain reliable results for most of the studied magnitudes.

The present study was designed because there is limited information available on short fasting times that would allow laboratories to produce reliable results and improve patient comfort during phlebotomy.

This study tries to assess if there are statistically significant differences between results obtained after a 10-hour fast and those obtained at 4 or 6 hours post-ingestion.

The first criterion considered was the non-exceedance of TEa. The only analyte that did not meet any TEa requirements (neither SEQC nor EFLM requirements) was TBIL. The rest of the analytes met at least the minimum TEa requirements set by the EFLM for both 4-hour and 6-hour fasting periods. However, it is important to highlight that this TEa criterion only takes into account the metrological characteristics of each technique (including imprecision and bias). Clinical laboratories employ a variety of analytical techniques, some of which are highly accurate or less so, depending on the technology available in the market. As a result, this leads to wider or narrower TEas.

The second criterion, based on the RCV, not only takes into account the metrological characteristics of each technique, but also the intra-individual biological variability (the phenomenon by which the values of biological analytes may vary within the same individual). This greatly enriches the characteristics of this requirement because in this study, differences in the results of certain analytes were evaluated in the same individual where an external factor may or may not have caused a change. All parameters evaluated in this study met this second criterion at both 4 and 6 hours.

The results obtained suggest we can reduce the fasting time to just 4 hours for the most of the magnitudes. Laboratories have to take into account that one of them only met RCV criterion, TBIL, and that others only met the minimum TEa, like CREA, Glc, P, TG and TSH. For this reason, each laboratory should stablish its own criterion; determine RCV and define which TEa is considered as acceptable. With those data each laboratory should decide if these magnitudes can be analysed after 4 or 6 hours fasting or not.

Our results are consistent with those obtained by Kus *et al*. (*6*). This study evaluates the effect of fasting on a limited number of biochemical markers (although these are the most commonly requested) and its strength lies in the number of participants (70,352). We believe that such a large sample provides strong validity to the results, which confirm that there are no statistically significant differences between fasting or not before undergoing a blood test.

On the other hand, our results are in disagreement with several studies cited in the literature (*7*-*12*). In our opinion, all of them present certain limitations, such as a small number of subjects (it should be noted that all these studies use between 17 and 33 volunteers, except for the study by Dong *et al.*, which includes 150 participants) and the use of biased populations in some cases (*8*). Furthermore, none of these studies take into account biological variability. In fact, all of them use statistical methods such as the t-test and Wilcoxon to assess the effect of fasting, without even considering the metrological variability. We believe that these limitations could reduce the reliability of their results.

As for the complete blood count, Kościelniak *et al.* studied the effect of a meal on those determinations at 1 and 2 hours post-meal. As we expected before starting our study, almost all the parameters were affected (*9*). The same happened to Dong *et al.*, who found differences in TSH concentrations at 2 hours after caloric intake compared to the baseline value (*8*). Therefore, we designed a study with fasting periods of 4 and 6 hours. We believe that this approach makes our study and its findings more representative of a real fasting situation.

We agree with Kjell Grankvist *et al.* that there are no national definitions of fasting for blood sampling (*13*). It is also evident that in most countries, multiple definitions of fasting are used, with varying durations and different instructions on what is allowed and what is not. Additionally, the number of analytes requiring fasting varies considerably between laboratories. Like them, we believe that glucose and triglycerides measurements should, if possible, be restricted to a limited period of the day. However, we disagree with the 12-hour fasting definition of the NORIP project. Based on our results, we propose a fasting period of 4 to 6 hours.

Our study also has some limitations worth noting. During the study period, certain confounding factors that could influence the results were not taken into account, such as physical activity, smoking, chewing gum, or the amount of water consumed between one blood test and the next.

Although the biological variation has been considered because it was necessary for calculating the RCV, the diurnal changes for the different parameters studied have not been taken into account. As seen by other authors in the bibliography, the circadian rhythm can have a significant impact on biochemical and hematological parameters. For example, the erythrocytes, leukocytes and platelets counts seem to be under clock gene control (*14*). Regarding biochemical magnitudes, some hormones have circadian rhythms, like TSH that shows higher concentrations during the night (*15*). Also for other magnitudes like iron, variations have been observed demonstrating concentrations peaks during sleep hours, either day and night sleep (*16*).

The authors are aware of this fact, but the study was conducted this way to replicate daily working conditions.

As the number of participants in the study was small (N = 30), it would be very interesting to have a larger number of participants in the study to increase the robustness of our results. Moreover, this would allow us to calculate an interval for the RCV, which would specify the maximum acceptable increase or decrease for each analyte.

The health status of the participants was not assessed because we were interested in having the most heterogeneous population possible, as this is what we will encounter in daily practice. For the same reason, the meal before 10-hour fasting was not controlled.

The most important strength of our study is that we have taken into account the biological and the analytical variation to decide whether fasting interferes in the laboratory results, while other authors only apply statistical tests in their works.

This study has demonstrated that results of acceptable quality can be obtained with only a 4-hour fasting period for the most of the magnitudes. This is very interesting as it could greatly improve the quality of life for certain groups of patients, such as diabetics, whose condition is heavily influenced by fasting hours. Some authors, concerned about these patient groups, have conducted studies to eliminate fasting in lipid profile analyses and have made recommendations accordingly (*17*, *18*). Anyway, authors recommend that each laboratory should establish its own criterion depending on their organization characteristics and attending population.

Although the recommended practice remains an overnight fast, this study presents a reliable alternative for those cases in which this condition cannot be met. Given the obtained results, which include the most commonly requested analytes by clinicians, there is a need for further research. Such research should include other analytes that are not as frequently requested but are essential to evaluate in order to continue improving the preanalytical phase and reduce fasting hours.

## Conclusion

The results obtained in this study demonstrated that a fasting period of 4 or 6 hours is sufficient to obtain reliable results. This could significantly improve the quality of life for patients undergoing analysis without compromising the quality of their results.

## Data Availability

The data generated and analyzed in the presented study are available from the corresponding author on request.
